# Supplementary material for: Cross-validation of meshless Navier–Stokes solvers in porous media flows
Source: Sci Rep. 2025 Sep 30;15:33837. doi: 10.1038/s41598-025-05272-x (PMC12484592; doi:10.1038/s41598-025-05272-x)
Supplement: Supplementary file 1 — Supplementary Material 1 [file 41598_2025_5272_MOESM1_ESM.pdf]

A MLBM and MNS algorithms comparison

MLBM algorithm

while the stopping criterion is not met **do**
    // COLLIDE
    for all Eulerian points x_i **do**
        for each lattice velocity c_k **do**
            Calculate f_k^{eq} (Eq. (14));
            Calculate f_k^{post} (Eq. (10));
        end for
    end for
    // STREAM
    for all Eulerian points x_i **do**
        for each Lagrangian point $x_i + \delta x_k$ **do**
            Interpolate $f_{k'}^{\text{post}}$ to $x_i + \delta x_k$ (Eq. (2));
            Overwrite $f_{k'}(x_i)$ with $f_{k'}^{\text{post}}(x_i + \delta x_k)$ (Eq. (8));
        end for
        Update ρ_{lb}, v_{lb} (Eq. (16));
    end for
end while

MNS algorithm

while the stopping criterion is not met **do**
    // UPDATE VELOCITY
    for all computational points x_i **do**
        Calculate intermediate velocity v' (Eq. (21));
    end for
    // PRESSURE-VELOCITY COUPLING
    for a predetermined number of iterations **do**
        for all computational points x_i **do**
            Update pressure (Eq. (22));
        end for
        for all computational points x_i **do**
            Update velocity with the new pressure gradient Eq. (23);
            Update C if required (Eq. (24));
        end for
    end for
end while

B Quantitative analysis of convergence

To provide a quantitative description of the convergence we calculate the grid convergence index (GCI) for permeability and drag coefficients, GCI_{k/d^2} and GCI_K respectively. GCI is calculated for the values of permeability or drag coefficient obtained on the finer discretization in each pair of subsequently refined grids. For example, if the pair of the discretizations $h_{\min} = 0.014$ and $h_{\min} = 0.01$ is considered, we calculate the GCI for the discretization $h_{\min} = 0.01$ and so on. For each such pair, GCI is calculated as

$$\text{GCI}_X = \frac{F_s|\varepsilon_X|}{r^{p_X} - 1} \tag{39}$$

where $r > 1$ is the ratio between the coarser and the finer $h_{\min}$ within the pair. p_X is the apparent order of convergence of the quantity X obtained from a linear regression fit to the $(\log(h_{\min}), \log(e_X(h_{\min})))$ data points where e_X is the relative error for a quantity X calculated as

$$e_X(h_{\min}) = \frac{|X(h_{\min}) - X(h_{\min} = 0.005)|}{X(h_{\min} = 0.005)}, \quad X \in \{k/d^2, K\} \tag{40}$$

and $F_s = 1.3$ is the safety factor. The relative error for quantity X in each pair, ε_X, is calculated as

$$\varepsilon_X = \frac{|X_{\text{coarser}} - X_{\text{finer}}|}{X_{\text{finer}}} \tag{41}$$

where subscripts *coarser* and *finer* denote the value obtained on the coarser and the finer of the two discretizations in the pair. We present the values of GCI in Tables 1–3. Due to the lack of data for MLBM at $r = 0.49$ for the coarsest discretization, we do not include the $h_{\min} = 0.02$ points in the calculation of GCI for both methods in general.

The GCI study confirms the observations from Fig. 6. If the highest values on the finest pair of discretizations for each method are considered, these are those for drag coefficient in $r = 0.062$ and $r = 0.49$ cases for MLBM ($7.2 \cdot 10^{-3}$ and $6.8 \cdot 10^{-3}$, respectively) and for drag coefficient at $r = 0.062$ and permeability at $r = 0.49$ for MNS ($3.9 \cdot 10^{-2}$ and $5.0 \cdot 10^{-3}$, respectively). This suggests that to obtain grid-independent results, one would need much denser discretizations in those setups.

h	N	GCI_{k/d^2}	GCI_K
0.014	70641	–	–
0.01	191802	7.9e-03	2.1e-02
0.007	555077	5.1e-03	1.1e-02
0.005	1515847	5.4e-03	7.2e-03

h	N	GCI_{k/d^2}	GCI_K
0.014	70785	–	–
0.01	191896	2.3e-03	8.9e-02
0.007	555333	4.8e-04	5.4e-02
0.005	1516144	7.0e-04	3.9e-02

Table 1. $r = 0.062$: Relative errors and GCI for permeability and drag coefficient. *Left:* MLBM results, *right:* MNS results. The MLBM permeability was for each simulation fitted in time with a function of the form $a + b\exp(-ct)$ and the asymptote a was taken to be the actual value of the permeability.

h	N	GCI_{k/d^2}	GCI_K
0.014	93348	–	–
0.01	247355	1.6e-03	2.0e-02
0.007	701396	2.0e-03	2.5e-03
0.005	1887779	3.2e-04	6.8e-03

h	N	GCI_{k/d^2}	GCI_K
0.014	93348	–	–
0.01	247355	5.4e-03	1.0e-02
0.007	701396	1.9e-03	4.8e-03
0.005	1887779	5.0e-03	4.7e-03

Table 2. $r = 0.49$: Relative errors and GCI for permeability and drag coefficient. *Left:* MLBM results, *right:* MNS results.

h	N	GCI_{k/d^2}	GCI_K
0.014	23792.0	–	–
0.01	60950.0	1.9e-02	1.3e-02
0.007	168569.0	8.1e-03	4.4e-03
0.005	445457.0	1.5e-03	1.0e-03

h	N	GCI_{k/d^2}	GCI_K
0.014	23767	–	–
0.01	60988	2.3e-02	4.2e-03
0.007	168704	6.3e-03	1.6e-03
0.005	445717	4.8e-03	1.4e-04

Table 3. $r = 0.6526$: Relative errors and GCI for permeability and drag coefficient. *Left:* MLBM results, *right:* MNS results.

C List of symbols used in the text

Greek letters	
β	compressibility parameter
δ	pointwise difference of the velocity field between the two solvers
δt	timestep length
δx	MLBM streaming distance
δ_i	local scaling factor for radial functions
δ_{ij}	Kronecker delta
$\lvert\Delta\rvert_{k/d^2}$	relative change of permeability in time
ε	refinement function shape parameter
ε_X	relative difference between the values of quantity X obtained on two subsequently refined discretizations
θ_s	polar angle on the obstacle's surface
λ	monomial weights vector
μ	dynamic viscosity
ν, ν_{lb}	kinematic viscosity, kinematic viscosity in LB units
ρ, ρ_{lb}	density, density in LB units
ρ_{ref}	reference density
σ_{ij}	stress tensor
τ	non-dimensional relaxation time
$\tilde{\phi}$	normalized signed distance function
ϕ_s	azimuthal angle on the obstacle's surface
ϕ_{sdf}	signed distance function
φ	porosity
Φ	radial function
ω_k	k-th lattice weight
Ω	volume of the fluid in the domain
**Latin letters**	
A	radial functions part of the approximation matrix or area of the solid walls
dA, ΔA	infinitesimal and finite surface element
b, c	radial functions and monomials part of the right-hand side vector of the approximation problem, respectively
c_s	lattice speed of sound
C	artificial speed of sound
C_{MLBM}	computational complexity of MLBM
C_{MNS}	computational complexity of MNS
d	computational domain's side length
D	dimensionality of the problem
e_k, $e_{k'}$	k-th discrete streaming vector, vector opposite to e_k

E	relative error of drag coefficient in the refinement parameters study
f_k, f_k^{eq}, f_k^{post}	k-th discrete velocity distribution function (VDF), equilibrium VDF, post-collisional VDF
$F_{H,i}$	i-th component of the hydrodynamic force acting on the obstacles
F_k	k-th discrete body force term
F_s	safety factor in grid convergence index calculation
g, g_{lb}	body force, body force in LB units
GCI_X	grid convergence index of quantity X
h	local internodal distance
h_{min}, h_{max}	minimal and maximal internodal distance
$\tilde{h}$	approximate internodal distance
I_b	set of boundary nodes indices
k	order of the polyharmonic spline radial function
k/d^2	dimensionless permeability
K, K_{ref}	drag coefficient, drag coefficient obtained on non-refined discretization
$\mathscr{L}$	linear differential operator
m	number of monomials augmenting the meshless approximation matrix
n	boundary normal direction
$\hat{n}_i$	local boundary normal vector
n_p	number of pressure correction steps
N, $N_{1,1}$	number of points in the refined and non-refined discretization
$\tilde{N}$	approximate number of nodes in the referenced works
N_b	number of the solid boundary nodes
N_L	stencil size in meshless approximation
N_p	number of monomials augmenting the approximation matrix
p	pressure
p_l	l-th monomial augmenting the approximation matrix
p_X	order of convergence of quantity X
P	monomial part of the augmented approximation matrix
P_i	i-th cross-section for calculating the mean x-component of velocity

List of symbols used in the text (1/2)

Latin letters (cont.)	
q	number of discrete velocities in the Lattice Boltzmann Method model or mean x-component of velocity
r	radial function argument or obstacle radius
S_i, $S_i(j)$	stencil of the i-th node, j-th member of S_i
t	time
Δt	time interval for calculating the relative change of permeability in time
u	approximated function
v, v_{lb}	macroscopic velocity, macroscopic velocity in LB units
v_{ref}	reference macroscopic velocity
Δv	absolute value of the difference of the velocity magnitude between MLBM and MNS
$w_{i,j}$	approximation weight of the j-th node in the i-stencil
x, x_i	space coordinate
x_d	de-queued node position in the node placing algorithm

List of symbols used in the text (2/2)
